# Aqueous seed extract of Syzygium cumini (asc) and polymeric nanoparticles (npasc) modulate adenine nucleotide and nucleoside hydrolysis in diabetic rats with candidiasis

**DOI:** 10.1186/1758-5996-7-S1-A2

**Published:** 2015-11-11

**Authors:** Paula Eliete Rodrigues Bitencourt, Carolina dos Santos Stein, Raphaela Maleski Borges, Maria Beatriz Moretto

**Affiliations:** 1 Universidade Federal De Santa Maria, Santa Maria, Brazil

## Background

Diabetes mellitus (DM) is an endocrine and metabolic disease that can cause alterations in platelet function[1]. Extracellular nucleotides such as ATP, ADP and adenosine are important in modulating processes linked to inflammation and thrombosis[2]. Diabetes-mediated changes in immune status may render patients more prone to fungal infections such as those caused by Candida spp[3]. The interest in developing nanostructured systems for improving the bioavailability and protection from physical and chemical degradation of herbal medicinal products has increased significantly.

## Objectives

The aim of the study was to evaluate the effect of the aqueous seed extract of Syzygium cumini (ASc) and of polymeric nanoparticles containing ASc (NPASc) on nucleoside triphosphate diphosphohydrolase (NTPDase), 5′-nucleotidase (5′NT) and adenosine deaminase (ADA) activity in platelets in a rat model of DM inoculated with Candida albicans.

## Material and methods

Male Wistar rats were divided into six groups (n=6): G1: control; G2: diabetic; G3: C. albicans; G4: diabetic+C. albicans; G5: diabetic+C. albicans+ASc; G6: diabetic+ C. albicans+NPASc. DM was induced by a single intraperitoneal (IP) injection of streptozotocin (60 mg/kg). C. albicans yeasts (105 UFC/mL) were inoculated (IP) in the respective groups after 15 days of diabetes induction. The treatment last for 21 days and ADA (U/L)4, 5′NT and NTPDase (nmol Pi/min/protein) 5 activities were measured in platelets. Ethic Committee number 074/2014.

## Results

We observed a statistical significant increase in ADA, 5′NT and NTPDase activities in G2 and G4 when compared to G1. Interestingly, these activities were also increased in G3. ASc and NPASc reverted this increase in ATP, ADP, and AMP hydrolysis and prevented the increase in ADA activity in G5 and G6, when compared to G4.

## Conclusion

Our results demonstrated that ASc and NPASc were able to act in the ATP dephosphorylating cascade and to protect cells and tissues during harmful conditions. These findings revealed the participation of purinergic signaling in pathophysiological situation of clinical relevance.

**Figure 1:**
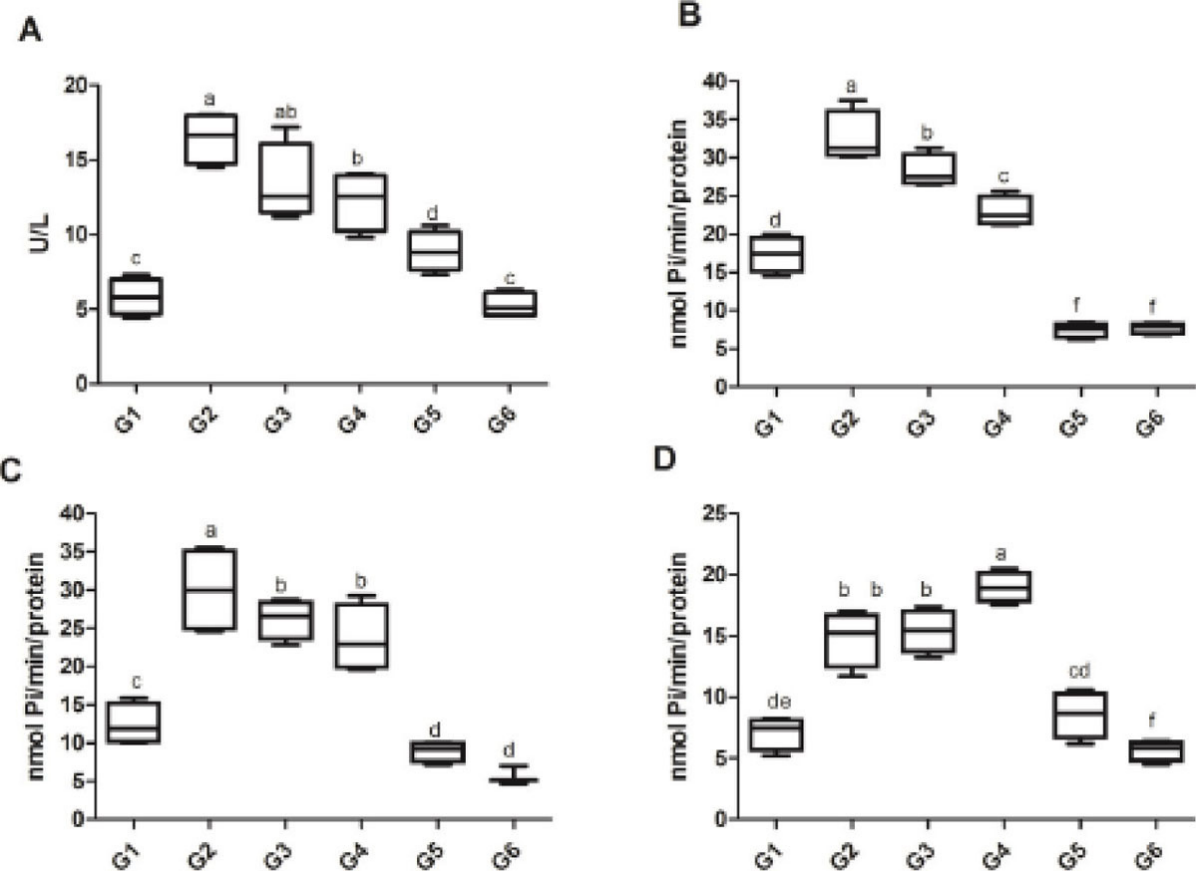
Effects of ASc and NPASc on ADA (A), 5′-nucleotidase using AMP as substrate (B) and NTPDase activities using ATP (C) and ADP (D) as substrate in platelets of diabetic rats with candidiasis. Data are mean±S.E.M. Groups with different letters are statistically different by ANOVA and Duncan post hoc test.
